# Supplementary material for: Complexome profiling of the Chlamydomonas psb28 mutant reveals TEF5 as an early PSII assembly factor
Source: Plant Cell. 2025 Mar 17;37(6):koaf055. doi: 10.1093/plcell/koaf055 (PMC12164590; doi:10.1093/plcell/koaf055)
Supplement: koaf055_Supplementary_Data [file koaf055_supplementary_data.zip › psb28_interactive.zip!/plots/intensity/qualityPlots/peptides/_Cre12.g537050.t1.2.html]

### Cre12.g537050.t1.2

Trivial name:   
SeqCoverage [median]: 38.80  
Mass: 7.33 kDa  
  
 

| Sequence | MissedCleavage | Mass [Da] | wtIntensity | muIntensity | Score |
| --- | --- | --- | --- | --- | --- |
| DLPSAPPSLK | 0 | 1023.56 | 8217.03 | 8517.90 | 87.64 |
| EVMAGIQAK | 0 | 945.50 | 20094.47 | 7876.63 | 120.65 |
| SLGEAFR | 0 | 778.40 | 8233.54 | 10690.59 | 120.65 |
|  |  |  | 36545.0 | 27085.1 |  |
